# Development of a core outcome set for psychological therapy trials on acute psychiatric inpatient wards

**DOI:** 10.1186/s12888-024-06294-x

**Published:** 2024-11-19

**Authors:** Pamela Jacobsen, Katherine Berry, Lucy Clarkson, Rebecca Hiscocks, India Hopkins, Ceri Morgan, Dhaarna Tandon, Ashley-Louise Teale, Natasha Tyler, Lisa Wood

**Affiliations:** 1 Department of Psychology, University of Bath, Bath, BA2 7AY UK; 2 Division of Psychology and Mental Health, School of Health Sciences, University of Manchester, Manchester, UK; 3 Manchester Mental Health NHS Foundation Trust, Manchester, UK; 4 NIHR Greater Manchester Patient Safety Translational Research Centre (PSTRC), Faculty of Biology, Medicine and Health, University of Manchester, Manchester, UK; 5 Division of Psychiatry, University College London, London, UK; 6 Acute and Rehabilitation Directorate, North East London NHS Foundation Trust, London, UK

**Keywords:** Inpatients, Consensus, Clinical Trial, Measures

## Abstract

**Background:**

Consensus on what outcomes should be included in trials of psychological therapies on acute psychiatric inpatient wards is currently lacking. Inclusion of different viewpoints, including service user perspectives, is crucial in ensuring that future trials measure outcomes which are meaningful and important. Development of a Core Outcome Set (COS), a minimum standardised set of outcomes to be measured and reported, would help improve synthesis and interpretation of clinical trial data in this area.

**Methods:**

Stage 1 of the COS development involved compiling a comprehensive long-list of outcomes from key sources including i) a systematic review of outcomes from published trials, ii) online survey of key stakeholders (service users, carers, healthcare professionals, researchers, and end users of research), iii) qualitative interviews with service users and carers. Stage 2 involved stakeholder groups short-listing the outcomes using consensus methods (e-Delphi survey). The final outcome set was derived from the short-list at a consensus meeting of stakeholders, facilitated by an Independent Chair.

**Results:**

A long-list of 68 outcomes was compiled from the systematic review (*n* = 30 trials), online stakeholder survey (*n* = 100 participants) and qualitative interviews (*n* = 15 participants). Fifty stakeholders took part in the e-Delphi study, where the long-list was cut down to a short-list of 12 outcomes over 2 rounds. Nine stakeholders took part in the final consensus meeting, and after some outcomes were removed and/or amalgamated, a final set of 6 outcomes was recommended for inclusion in the COS. These were *Ability to Cope*, *Hopefulness, Quality of Life, Psychosis Symptoms, Mood,* and *Self-Harm Behaviours.*

**Conclusions:**

Widespread future adoption of the COS will reduce research waste by ensuring that outcomes are more easily comparable across trials, and that the full range of stakeholder priorities are represented in trial outcomes. This makes it more likely that effective therapies will be identified in a timely fashion and successfully implemented in routine clinical practice. The final 6-outcome COS should be feasible to implement given the need keep participant burden to a minimum in inpatient trials. Further work is needed to make recommendations for the best outcome measurement instruments to use, including the use of patient-reported outcomes alongside clinician-rated measures.

**Trial registration:**

Not applicable.

**Supplementary Information:**

The online version contains supplementary material available at 10.1186/s12888-024-06294-x.

## Introduction

Randomised controlled trials are used to assess the efficacy and effectiveness of healthcare treatments. However, many trials do not translate into benefits in patient care due to serious limitations in their conduct and reporting [1]. A key limitation relates to inadequate selection and reporting of outcome measures. One initiative to address this is the development of core outcome sets for clinical trials. Core outcome sets (COS) are an agreed, standardised collection of outcomes which should be measured and reported, as a minimum, in all trials for a specific area of clinical research [2].

There are two main steps to developing a COS. The first step is to set out ‘*what*’ should be measured (also known as outcomes, or outcome domains). An example of an outcome would be depression. The second step is to then specify options for ‘*how*’ an outcome should be measured (also known as an outcome measure instrument (OMI)). An example of an outcome measure instrument for the outcome of depression would be the Patient Health Questionnaire (PHQ-9) [3]. Note that there may be severable suitable outcome measure instruments for the same outcome. The goal of developing a COS is always to firstly address the question of what should be measured, rather than how it should be measured. This is particularly important to understand in relation to gold practice in COS development, which is that all outcomes must be potentially considered for inclusion *regardless of the current OMIs available to measure them* [4]. This guards against potentially important outcomes being overlooked because of a lack of a validated tool to measure them. Identification of core outcomes can be an important catalyst to the development of validated and psychometrically robust tools to measure important outcomes which may have been neglected in earlier literature, particularly in relation to outcomes of particular importance to service users and carers (e.g. recovery measures in mental health such as the Questionnaire about the Process of Recovery (QPR) [5].)

The development and implementation of core outcome sets promotes meaningful interpretation and synthesis of studies, by reducing heterogeneity in outcome measurement. This is important because there are a wide range of potential outcomes which could be measured in a clinical trial. However, the widespread use of non-overlapping outcome measures between trials increases research waste and limits the progression of the evidence base through synthesis methods such as meta-analyses, which rely on comparable outcomes being measured across trials in the same area. Despite advancements in COS development, the latest systematic review of available COS in 2021 reported that only 5/370 (1.4%) were for mental health trials, with 350/370 (94.6%) being for physical health trials [6]. This indicates a clear need for more mental health core outcome sets to be developed.

Another key function of a COS is to widen the range of viewpoints which are considered in the selection of outcome measures. Core outcome sets are developed by consensus methods which ensures that all key stakeholders, including service-users and carers, are involved in selecting outcomes to be included in the COS. This is important as different stakeholders can have different priorities for treatment outcomes. For example, service users may emphasise quality of life and overall functioning, rather than a sole focus on symptom reduction [7]. Including service user views on outcome measures in mental health is particularly important due to high levels of stigma associated with mental health diagnoses [8], particularly for people with severe mental illness [9, 10]. Societal prejudices against marginalised groups such as mental health service users can lead to their views not being sought out in the first place due to negative stereotypes, or their views being discounted and ignored due to a lack of perceived credibility, which is a form of epistemic injustice [11, 12].

Inpatient care is an important area of mental health services where consensus on outcome measurement is currently lacking. Acute inpatient care is for people with complex needs and high levels of risk, with diagnoses including schizophrenia, bipolar disorder, and personality disorder. Globally, inpatient care accounts for a disproportionate amount of healthcare budgets [13]. However, service user and carer experiences of an inpatient admission are often highly negative [14–16], and high short-term readmission rates [17–19] indicate failures in the effectiveness of inpatient care in helping people to stay well after discharge. Although models of inpatient care differ across international healthcare systems, in general the key components are assessment and treatment of acute illness, management of risk, and provision of a safe environment for rehabilitation [20]. To fulfil these functions, inpatient care requires a highly skilled multi-disciplinary team delivering a full range of psychosocial interventions. However, access to evidence-based inpatient psychological therapies is limited in the UK [21], and the international literature on inpatient care further indicates a lack of access to therapies as a common reason for service user dissatisfaction with care [22].

Developing the evidence base for inpatient psychological therapies, which are appropriately adapted to the setting, and specifically focused on the therapeutic targets of acute care (e.g. crisis resolution) is therefore an urgent priority. A newly developed core outcome set will help better align the therapeutic targets of future trials with the expressed needs of services users and carers, alongside the views of researchers and healthcare professionals. Related core outcome sets which have already been developed include one for evaluating interventions designed to improve discharge from inpatient services [23]. This COS included four outcomes; *readmission, quality of life, suicide completed*, and *service-user reported psychological distress* [23]. The COS did not include the additional step of specifying which outcome measure instruments should be used to measure these outcomes, noting that this is an important step for further work. Whilst complementary to the aims of the current study, the scope and intended application differed in that it focused on the transition from inpatient to community services, rather than treatment delivered during the inpatient admission itself. Furthermore, the discharge COS was designed for a wider range of interventions, including at a service level (e.g. changing discharge procedures), not just psychological interventions. As noted above, existing mental health COS are very limited, and previously developed COS for severe mental illness (psychosis and schizophrenia) have focused on evaluation of services [24] and patient-reported outcomes [25] in community rather than inpatient settings. The COS of patient-reported outcome measures in psychosis for community services comprised 9 outcomes across different domains (*depressive symptoms, positive psychosis symptoms, negative psychosis symptoms, manic symptoms, personal recovery, sleep disturbance, functioning, physical health, and anti-psychotic medication side effects*) [25]. This shows the importance of having a wide range of stakeholder input to develop a balanced and comprehensive COS, which is not solely focused on symptom reduction, but also includes other important outcomes such as recovery and functioning.

The research objective of the current study was therefore to develop a Core Outcome Set (COS) for psychological therapy trials conducted in acute mental health inpatient services. The population to be covered by the COS was defined by the setting in which people were receiving care (acute mental health inpatient wards) rather than a specific diagnosis or health condition, as people admitted to mental health wards have a range of different difficulties and diagnoses (e.g. psychotic symptoms, mania and/or depression, suicidal ideation, self-harm). The interventions to be covered by the COS were defined as psychological (‘talking’) therapies, with no restriction on the type of therapy model used (e.g. cognitive-behavioural therapy) or mode of delivery (e.g. group/individual). The COS was developed to cover therapies primarily designed to be delivered either entirely or primarily within the course of an inpatient admission, rather than primarily community-based therapies which may be initiated during an inpatient admission. The aim of this study was to develop the ‘what’ of the COS, i.e. what outcomes should be measured. Specifying ‘how’ these should be measured i.e. what outcome instruments should be used was out of scope and will be covered in future work.

## Method

We used best-practice methodology for COS development, according to the COS-STAD recommendations, which stipulates that key stakeholders should be involved in the COS development, including healthcare professionals, researchers, and service users/carers [4]. The COS-STAD guidelines further recommend that the initial list of outcomes must include, at minimum, both healthcare professionals and service user/carer views, and there must be a transparent a priori consensus method including criteria for including, dropping, and adding outcomes over the stages of the COS development. In line with these guidelines, our method for developing the COS comprised three main stages.

1. Conducting a systematic review of existing literature and consulting with key stakeholders through an online survey and semi-structured interviews, to develop a long list of possible outcomes.
2. Using e-Delphi methodology to generate consensus and refine the longlist into a shortlist of outcomes.
3. Finalising the core outcome set from the shortlist at a consensus meeting.

### Protocol/registry entry

The protocol was pre-registered before data collection began on the COMET (Core Outcome Measures in Effectiveness Trials) website (https://comet-initiative.org/Studies/Details/2045) and on the Open Science Framework (10.17605/OSF.IO/ENY8G). This paper is written in line with reporting guidelines for COS development (COS-STAR; [26]).

### Research team reflexivity and PPE (people with personal experience involvement)

The research team included clinical academics, a research psychologist, trainee clinical psychologists, psychology MSc students, and a lived experience researcher. As a team we brought multiple perspectives and experiences to the COS development, based on working in inpatient settings as healthcare professionals, conducting extensive inpatient research, and having lived experience as an inpatient service user. We recognised that these multiple experiences informed our view of outcomes in inpatient care. For example, we reflected on how people receiving inpatient care are often a highly marginalised group in society, and historically their experiences and narratives may have been ignored or discounted in terms of treatment outcomes. We were therefore particularly aware of the need to recognise and address these power imbalances in the process of short-listing outcomes. Alongside the lived experience researcher as a member of the core research team, we additionally consulted with an advisory group of service users and carers from the University of Bath at the beginning of the project which helped to inform the design and conduct of the study.

### Participants

We identified 5 key stakeholder groups for the COS development process, and they were drawn upon at relevant stages, which is outlined further below: -

1. Service user: Anyone who is currently, or has in the past, been in receipt of inpatient care from mental health services (National Health Service (NHS) and/or private) within the last 5 years in a UK setting.
2. Informal carer: Anyone who self-defines as a non-professional carer of someone in receipt of inpatient care from mental health services (NHS and/or private) in a UK setting (currently, or in the past five years).
3. Healthcare professionals: Anyone who has been a patient-facing healthcare professional based on an inpatient ward, not limited to those who provide psychological therapy, but those who provide other elements of inpatient care for someone receiving mental health care (NHS and/or private) in a UK setting (currently, or in the past five years).
4. Researchers: Anyone who is a researcher or trialist of mental health care (NHS and/or private) in a UK setting (currently, or in the past five years).
5. End users of research: Anyone who is a key informant including: policy makers, non-government organisations, National Health Service management, commissioners, mental health advocates and third sector/governmental professionals (such as social workers) of mental health care (NHS and/or private) in a UK setting (currently, or in the past five years).

For the semi-structured interviews with service users and carers, we additionally restricted the experience of inpatient care to within the last year (rather than 5 years) so that people would be more easily able to recall their experiences in detail.

We recognised that people often have multiple identities (for example, someone may have lived experience as both a service user and a healthcare professional), and therefore we asked people to decide which stakeholder group they would like to primarily respond as for each stage. We restricted participation to people with UK-related experience because health care services can vary widely between different countries due to various factors (e.g. funding models), and the aim was to produce a COS which would be a good fit for the UK NHS (National Health Service). However, we did plan to include eligible international studies in the systematic review, to improve the generalisability and scope of the final COS. Participants were recruited using a variety of sources, including adverts disseminated via social media (e.g. Twitter, Facebook), mental health charities (e.g. MQ participate), and professional bodies (e.g. British Psychological Society).

### Stage 1 – long-listing

#### Systematic review

First, we conducted a systematic review of existing studies of psychological therapies in acute inpatient mental health settings to extract outcome measures. The review protocol was written and registered on a publicly accessible registry (https://www.crd.york.ac.uk/prospero/display_record.php?ID=CRD42022345003).

We first screened studies from three existing systematic reviews of inpatient psychological therapies, which between them covered a range of study designs and therapeutic approaches [27–29]. We then ran additional electronic database searches (PubMed and Scopus) on 1st June 2022 to identify any new studies which had been published since the last review was conducted. See Additional File 1 for full search terms.

Studies were eligible for inclusion if they were randomised controlled trials (RCTs) of a psychological therapy (any type) on acute inpatient wards (regardless of participant diagnosis). All records were independently double-screened for inclusion by two researchers at both title/abstract and full-text review. Any conflicts were resolved by discussion, with the senior author (PJ) being consulted where necessary. Data extraction was completed using a standardised data extraction template, with all data independently double-extracted by two researchers with any conflicts resolved by discussion. We extracted data on what types of outcomes, what time-points outcomes were measured at, and how outcomes were measured (outcome measure instruments). All stages of the review were conducted using the review software package Covidence.

#### Online stakeholder survey

We recruited participants from all 5 stakeholder groups to take part in an anonymous online survey asking what outcomes would be important to measure for inpatient psychology trials (hosted via the online platform Qualtrics). The questions were based on a previous COS development study related to psychiatric inpatient settings [23] and in collaboration with the University of Bath Lived Experience panel. We included a mixture of general questions addressed to all stakeholders and questions tailored to specific groups (see Additional File 1 for survey questions).

#### Qualitative interviews with service users/carers

We focused our in-depth qualitative data collection with service users and carers, given that their priorities for outcomes were least likely to be already captured in the existing literature. Qualitative interviews also allowed for deeper exploration of meanings in the outcomes service users and carers suggested, as we were also aware this stakeholder group will refer to outcomes using different words and phrases compared to researchers and healthcare professionals who will be more likely to use medical terminology (e.g. feeling low vs. depressive symptoms). The semi-structured interview topic guide was based on the survey questions for service users and carers, with the aim of exploring participants responses in more detail (see Additional File 1 for topic guide). The interviews were conducted online using video calls by CM and RH and were recorded for verbatim transcription. None of the interviewees were previously known to the interviewers in any capacity. CM developed the initial coding framework after a period of deep immersion in the data through re-listening to interview recordings, and transcribing, checking, and re-reading transcripts. Initial codes were then discussed and further refined in the wider team with RH, PJ, LC, and IH.

#### Development of long-list

Outcomes from the systematic review, online stakeholder survey, and the qualitative interviews were first pooled together. Duplicates were then identified by mapping verbatim outcomes onto standardised terms to group them together (e.g. “being released” mapped onto “discharge”). This was an iterative process done by discussion and consensus by the core research team (PJ, LC, IH, & CM). Where there was uncertainty as to whether two similar terms were referring to the same outcome, we erred towards keeping them separate as to keep a finer degree of granularity in the data in the initial stages. We then mapped each outcome to an established taxonomy of outcomes across different health areas [30]. This taxonomy has five core areas (death, physiological/clinical, life impact, resource use and adverse events) further divided into 38 more detailed outcome domains under each core area.

The final long-list of outcomes was then used to populate the e-Delphi survey. The wording of all outcomes was checked by our lived experience researcher and University of Bath Lived Experience panel to ensure they were easily understandable and written in plain English.

### Stage 2 – short-listing

#### Procedure

The e-Delphi survey was hosted by the COMET Delphi Manager software (http://www.comet-initiative.org/delphimanager/) specially designed for core outcome set development. Over 2 rounds, participants from across stakeholder groups rated each long-listed item on a 9-point Likert scale, grouped into 3 categories (‘Not important’ – 1–3, ‘Important but not critical’ – 4–6, ‘Critical’ – 7–9). We originally planned that any outcome scored as ‘Critical’ (7 – 9) by ≥ 75% of participants would automatically be short-listed, and any outcome scored as ‘Not important’ (1–3) by ≥ 75% of participants would automatically be excluded after round 1. However, we later refined these criteria as no outcomes satisfied these criteria after round 1 (see results section for further detail). All other outcomes would then be presented again in round 2 for re-rating, with participants shown a visual summary of how others in each stakeholder group voted in round 1. Participants were given the option of suggesting any additional outcomes they felt were important in stage 1 using a free-text box. Any outcomes scored as ‘Critical’ (7 – 9) by ≥ 75% of participants after round 2 would meet criteria for the short-list.

### Stage 3 – final consensus meeting

#### Procedure

An online consensus meeting was held to decide which outcomes to include in the final COS, from the short-list. Participants were recruited across key stakeholder groups. The consensus meeting was chaired by an independent researcher with expertise in consensus methodology and inpatient research who was not a member of the core research team. Participants were presented with the short-listed outcomes in advance of the meeting and given an overview of the aims of the meeting. Given the power imbalances which may arise both within and between different stakeholder groups, the importance of everyone’s viewpoint was emphasised. It was acknowledged that different people may prioritise different outcomes to include in the final COS, but the goal of the meeting was to reach consensus on the overall most important outcomes to include. Participants were further instructed that limitations to measurement of outcomes (e.g. current lack of psychometrically validated outcome measure instruments) should not be a barrier to inclusion in the final COS. This was because if an outcome was judged to be important to include this could provide the impetus for better measure development in the future. Use of anonymous online voting software was used during the meeting to help facilitate the process of reaching consensus.

## Results

**Fig. 1:**
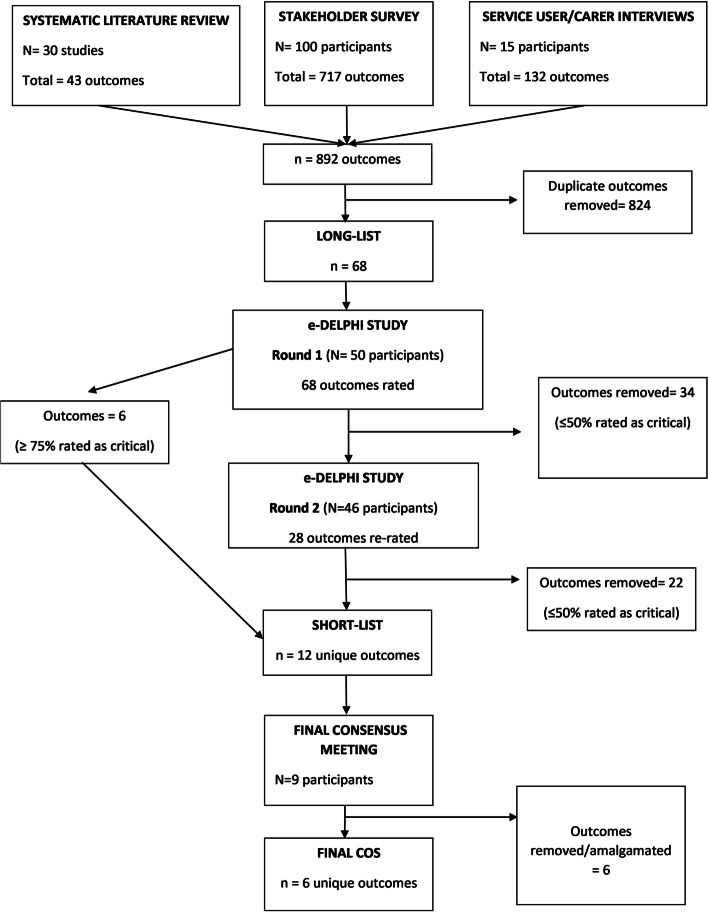
Summary of COS development

Outcome selection across all stages of the COS is summarised in Fig. 1.

### Stage 1 – long-listing (systematic review, online survey, qualitative interviews)

For the systematic review, a total of 1952 studies were screened for eligibility, including studies identified via previous reviews, and updated searches of electronic databases. Sixty-two studies were assessed for eligibility at the full-text stage, with 30 meeting inclusion in the final sample (see Additional File 1 for PRISMA diagram).

Trials were conducted across a range of countries, although most were from the United Kingdom (*n* = 8) and United States (*n* = 6) (see Additional File 1 for table of characteristics and full reference list for included studies). Only 13/30 studies (43%) clearly designated a primary outcome, so we extracted data on all reported outcomes, regardless of designation of primary/secondary outcomes. There were 152 outcomes in total across all 30 studies (range = 2–14, mean = 5.5). After grouping together duplicate outcomes described using different terms, there were 43 unique outcomes across trials.

One hundred people took part in the online survey (Service Users = 33, Carers = 10, Healthcare Professionals = 31, Researchers = 18, End Users of Research = 8). Most respondents were women (79/100, 79%), and of White ethnicity (83/100, 83%). There was a spread of age-groups (18–29 years = 24/100;24%, 30–39 = 26/100;26%, 40–49 = 26/100;26%, 50–59 = 13/100;13%, 60 + = 11/100;11%). An initial total of 717 outcomes were identified from verbatim survey responses.

Fifteen participants (14 Service Users, and 1 Carer) took part in individual qualitative interviews conducted online via videocall. There were 4 men and 9 women, with a spread of ages from 18 to 49. There were a range of ethnicities (White = 4, Black/Mixed ethnicities/Other = 8, prefer not to say/missing = 3). One hundred and thirty-two outcomes were coded from the interview transcripts. Analysis of the interview data is reported in more depth in a linked qualitative paper [31].

Collating together the outcomes from the systematic review, the online survey, and the qualitative interviews resulted in a total of 892 outcomes. After grouping together the same outcomes referred to under different terms, 824 duplicates were identified and removed. This resulted in 68 different outcomes on the long-list, which we further categorised using an established taxonomy of outcomes [30] as described in the method (see Table 1 for longlist and shortlist of outcomes).

**Table 1:** Longlist & shortlist of outcomes (grouped by outcome domain)

Longlist (68 outcomes)	Shortlist (12 outcomes)
**Domain: Adverse events**	
1. Verbal/physical aggression	
2. Use of restrictive practices on ward e.g. use of restraint/seclusion	
3. Self-harm	1. Self-harm
4. Suicidal thoughts	2. Suicidal thoughts
**Domain: Cognitive Functioning**	
5. Ability to think clearly	
6. Concentration	
7. Sense of humour	
8. Memory	
9. Problem solving	
**Domain: Emotional Functioning/Wellbeing**	
10. Ability to cope	3. Ability to cope
11. Feeling like you can make decisions for yourself	4. Feeling like you can make decisions for yourself
12. Feeling you can manage your emotions	5. Feeling you can manage your emotions
13. Enjoyment in activities	
14. Hopefulness	6. Hopefulness
15. Happiness	
16. Shame	
17. Impulsivity (acting on the spur of the moment)	
18. Having a clear sense of identity	
19. Feeling motivated to pursue your goals	
20. Feeling like life has a purpose	
21. Feeling like you have a purpose	
22. Self-compassion	
23. Feeling capable	
24. Feeling confident in yourself	
25. Self-esteem	
26. Feeling internalised stigma (self-stigma)	
27. Feeling stigmatised by others	
28. Stress	
29. Understanding of difficulties	
30. General wellbeing	
31. Doing things I enjoy	
**Domain: Social Functioning**	
32. Ability to socialise with others	
33. Ability to communicate needs and wishes	7. Ability to communicate needs and wishes
34. Ability to communicate thoughts and feelings	
35. Feeling connected to other people	
36. Feeling part of society	
37. Feeling part of your community	
38. Feeling isolated	
39. Doing things to serve your community/others	
40. Being able to see things from other people’s perspectives	
41. Recognising support from others	
42. Living life in line with values	
**Domain: Role Functioning**	
43. Taking charge of things for myself	
44. Being able to fulfil my voluntary/community roles	
45. Being able to fulfil my role at work	
46. Being able to fulfil my role with family members/friends/pets	
47. Being able to manage my money/finances	
48. Being able to manage things at home e.g., cooking, cleaning	
**Domain: Physical Functioning**	
49. Personal care e.g., showering, wearing clean clothes, grooming	
50. Taking care of physical health conditions e.g. asthma, diabetes	8. Taking care of physical health conditions e.g. asthma, diabetes
51. Sleeping well	
52. Taking care of daily health e.g., regular exercise, balanced meals	
**Domain: Life Impact**	
53. Following treatment plan	
54. Satisfaction with treatment	
55. Quality of life	9. Quality of life
**Domain: Resource Use**	
56. Frequency of crisis episodes after discharge	
57. Level of care needs during admission e.g., level of 1:1 nursing observation	
58. Level of care needs after discharge	
59. Leave from ward	
60. Length of admission	
61. Readmission to hospital	
62. Use of other services e.g., A&E, GP, community MH services	
**Domain: Psychiatric Outcomes**	
63. Anxiety	10. Anxiety
64. Depression	11. Depression
65. General distress	
66. Mania e.g. elated mood, irritability	
67. Psychosis e.g. hearing voices, paranoia, grandiose beliefs	12. Psychosis e.g. hearing voices, paranoia, grandiose beliefs
68. Substance misuse	

### Stage 2 – short-listing

Fifty people took part in the e-Delphi study (Service Users = 15, Carers = 5, Healthcare Professionals = 17, Researchers = 12, End Users of Research = 1). As with the survey, the majority were women (36/50; 72%) and of White ethnicity (38/50; 76%). There was a spread of age-groups (18–29 years = 8/50;16%, 30–39 = 11/50;22%, 40–49 = 12/50;24%, 50–59 = 5/50;10%, 60 + = 5/50;10%). There were missing demographic data for 9 participants due to a technical error on the software platform. All 50 participants responded to round 1 (68 outcomes presented for rating), and 46/50 (92%) responded to round 2.

After round 1, 6/68 outcomes were rated as ‘Critical’ (7–9) by ≥ 75% of participants and were automatically put forward to the short-list as per protocol. However, as per our pre-specified protocol criteria, no outcomes were scored as ‘Not important’ (1–3) by ≥ 75% of participants at this stage. Therefore, we made a protocol amendment and adjusted the threshold to ≤ 50% rating as ‘Critical’ which led to 34 outcomes being removed. In round 2, 28 outcomes were re-rated by participants. A further 22 outcomes were further removed after this stage using the amended criteria (≤ 50% rating as ‘Critical’). This resulted in 12 outcomes being put forward to the final consensus meeting (see Table 1).

### Stage 3 – final consensus meeting

Nine people took part in an online consensus meeting, chaired by an independent person outside of the research team. Participants were 3 Service Users, 2 Researchers, 1 Carer, and 3 Healthcare Professionals (8/9 (89%) women, 8/9 (89%) White ethnicity). Participants ranked the 12 short-listed outcomes according to importance using anonymised voting software at the beginning and end of the meeting to give an overview of consensus within the group. Overall, there were no areas of strong disagreement, with no definite number of outcomes recommended, but with an initial top 6 clearly prioritised. The group suggested that two of the short-listed outcomes were very similar and could be amalgamated (incorporating ‘*feeling you can manage your emotions’* under ‘*ability to cope’* which was seen as a broader and more helpful outcome). Furthermore, *‘anxiety’* and ‘*depression*’ were seen as important, but would be better included under an umbrella outcome of ‘*mood*’ which was seen as more inclusive and generalisable across different problem areas. There was also a discussion over the complexities of defining the meaning of ‘*self-harm’*. It was suggested the outcome should focus on self-harm ‘*behaviours*’, as these related to risk behaviours which often trigger the need for an inpatient admission such as cutting or taking an overdose, and reduction of risk is a key focus of inpatient care.

**Fig. 2:**
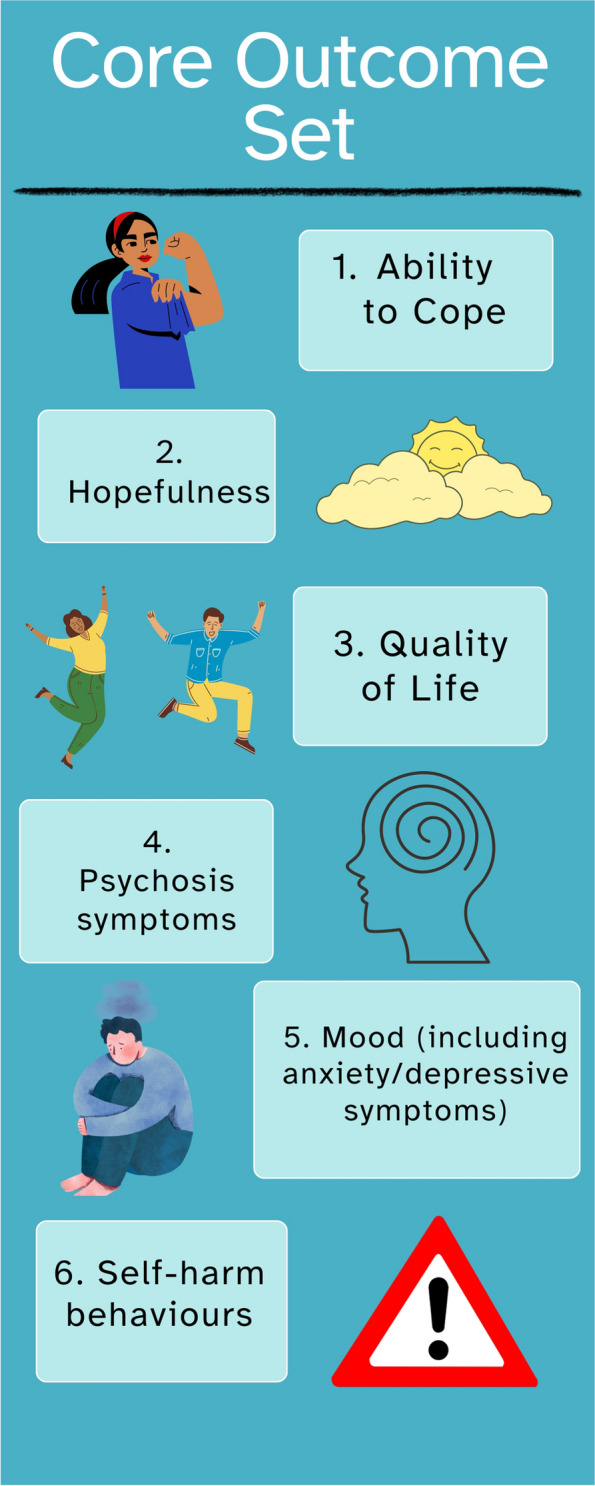
Final Core Outcome Set

After the final consensus meeting, and amalgamating outcomes where suggested, a final set of 6 outcomes were included in the Core Outcome Set. A range of different domains were covered in the final set, including 2 outcomes under the Emotional Functioning/Wellbeing domain (*Ability to Cope* and *Hopefulness)*, 1 under Life Impact (*Quality of Life*), 2 under Psychiatric Outcomes (*Psychotic Symptoms* and *Mood*) and 1 under Adverse Events (*Self-harm Behaviours*). See Fig. 2 for the final Core Outcome Set.

## Discussion

The aim of this study was to develop a Core Outcome Set (COS) for psychological therapy trials on acute psychiatric inpatient wards. We developed the COS by reviewing the existing trials literature and consulting with key stakeholder groups (service users, carers, healthcare professionals, researchers, and end users of research). Consensus methods were used to create a short-list of outcomes which was then further refined into a final COS of 6 outcomes; *Ability to Cope*, *Hopefulness*, *Quality of Life*, *Psychosis Symptoms*, *Mood*, & *Self-harm Behaviours*.

As expected, the outcomes in the COS overlap to a certain degree to other COSs in related areas. The Tyler et al. COS for discharge from inpatient care [23] also included *Quality of Life* as an outcome, as do a large majority of COS across different health areas [6]. This shows the importance of holistic outcomes which take into account a person’s overall life and wellbeing and how it may be impacted by a particular health condition. In comparison to the COS for psychosis community services, both include outcomes relating to a range of different domains including symptoms, recovery, and functioning [25], although the community COS has more outcomes overall (9 vs. 6 respectively). We did not pre-determine, or otherwise restrict the COS to a fixed number of outcomes, or domains which must be covered. However, given the final COS ended up including a range of different domains, including functioning and life impact alongside symptoms and adverse events, we believe this represents a balance of outcomes prioritised by key stakeholders, in line with the general goal of core outcome set development.

### Strengths and limitations

The strengths of our approach included a strong emphasis on service user involvement throughout the planning, conduct, and analysis of the study, alongside having service users as participants in the study. We attempted to compensate for the under-representation of service user and carer views in the existing literature, by making them the focus of our more in-depth qualitative interviews. Our sampling strategy focused on recruitment routes outside of clinical services, which arguably may have broadened the range of views included in the study, by not limiting participation to people currently in receipt of mental health services. Avoiding recruiting participants through clinical teams can also guard against unconscious bias and excessive gate-keeping [32]. The converse to this is that we do not have detailed information on the nature of people’s care with services, diagnoses, or treatment plans, and so we cannot explore how these factors may have impacted people’s views. Likewise, we did not ask healthcare professionals to report their core professional group, so we are also unable to assess whether there was a balance of representation across nurses, psychologists, psychiatrists etc. or how views may have differed between these sub-groups.

Although all key stakeholder groups were represented across all stages of the study, we did not impose quotas for each group, which meant that we ended up with uneven proportions of different groups across stages. Notably, we only had 1 person who identified as an end user of research in the e-Delphi stage. We found recruiting this stakeholder group particularly challenging through non-NHS routes as we did not have permission to directly approach Integrated Care Boards (ICB), who include commissioners and other end users. This could be addressed in future mental health core outcome sets by broadening recruitment pathways to target all stakeholder groups more efficiently. It is also important that the people involved in developing the COS reflect the intended target population. For example, Black people are over-represented in inpatient care compared to the general population, and are four times more likely to be subject to compulsory detention under the Mental Health Act compared to White people [33]. Although the participant sample overall was ethnically diverse, most respondents across all stages and stakeholder groups identified as White British. Targeted recruitment strategies could have been valuable in purposively sampling people from Black ethnic groups to increase the representation of their views in the COS.

### Implementation and future research

In developing a COS which was designed to be implemented in an acute inpatient setting, we were particularly aware of the need for a relatively small outcome set, which could be feasibly delivered in a short period of time reducing administrative burden. Minimising participant burden is also particularly important for inpatient therapy trials as participants may be experiencing high levels of distress and impairment associated with symptoms, including reduced concentration levels and cognitive difficulties [34]. Recommending specific Outcome Measure Instruments (OMI) for each outcome was out of the scope of the current study, therefore it is not possible to specify the expected average time to complete the COS. However, as a range of OMIs are available for each outcome, which vary according to the length of the measure and how long they take to complete, this allows for extra flexibility in making the COS as quick and simple to complete as possible within the constraints of the available resources of each individual trial.

It is important to emphasise that a COS is a minimum, not a maximum, set of outcomes to be measured and reported in a trial. Therefore, psychiatric inpatient trials of psychological therapies can implement the current COS, alongside adding any additional outcomes which would be specifically relevant to the treatment target. For example, previous inpatient therapy trials have included measures of self-stigma [35] and sleep dysfunction [36] as primary clinical outcomes in line with the goals of the therapy being evaluated. A COS is no threat to the tailoring of outcomes to the intended therapeutic target, and also does not dictate which outcomes should be designated as primary or secondary. This is an important issue to address within the research community, given that a recent survey of triallists across different health areas cited the preference for researchers to choose their own outcomes, or the idea that core outcome sets restrict the choice of outcomes, as major attitudinal barriers to the uptake of core outcome sets [37]. Recent adoption of guidelines by major UK funders such as the NIHR (National Institute for Health Research) in encouraging the use of available core outcome sets in grant applications is a welcome initiative which helps to raise awareness of the availability of relevant COSs as well as embedding the expectation that they should be used as part of standard practice.

Further work will be needed to examine how this COS might be applied in international settings beyond the UK, and also in a range of different inpatient settings beyond acute wards, including rehabilitation, forensic, and specialist wards (e.g. eating disorder units). This could involve further work with stakeholders on how the COS may need to be further developed to specify any variations to make it more widely applicable, including the adding or removing of any outcomes for these other settings. As already discussed, the specification of Outcome Measure Instruments (OMIs) for each outcome was out of scope of the current study, so there is a need for future work to set out valid and reliable OMIs for each outcome. This should include a consideration of the use of both self-report and clinician rated measures, alongside outcomes which can be extracted from Electronic Health Records (EHRs) such as using risk/incidence reports for outcomes such as self-harm behaviours.

## Conclusions

We have developed a Core Outcome Set (COS) for use in trials of psychological therapies on acute psychiatric inpatient wards, comprising six outcomes; *Ability to Cope*, *Hopefulness, Quality of Life*, *Psychotic Symptoms*, *Mood* and *Self-harm Behaviours*. The COS was developed with the input of a range of stakeholders, and so the inclusion of these outcomes in future trials should ensure that trials measure and report outcomes which are meaningful and important. The COS represents a minimum set of outcomes to measure to facilitate future data synthesis across trials, whilst affording trialists the flexibility to add additional outcomes according to researcher choice and in line with the specific therapeutic targets of the intervention.

## Supplementary Information

Additional file 1. 1) Search teams for systematic review. 2) Online survey questions. 3) Qualitative interview topic guide. 4) PRISMA diagram – selection of studies for systematic review. 5) Table of characteristics for studies included in systematic review. 6) Reference list for studies included in systematic review.

## Data Availability

The datasets generated and analysed during the current study are available on the Open Science Framework (10.17605/OSF.IO/ENY8G) and in the University of Bath Research Data Archive (10.15125/BATH-01265).

## Abbreviations

COS: Core Outcome Set
COS-STAR: Core Outcome Set–STAndards for Reporting
EHRs: Electronic Health Records
ICB: Integrated Care Board
NHS: National Health Service
OMI: Outcome Measure Instrument
PPE: People with Personal Experience involvement
PROM: Patient Reported Outcome Measure
RCT: Randomised Controlled Trial
UK: United Kingdom
