# Supplementary material for: Development of a core outcome set for psychological therapy trials on acute psychiatric inpatient wards
Source: BMC Psychiatry. 2024 Nov 19;24:821. doi: 10.1186/s12888-024-06294-x (PMC11575046; doi:10.1186/s12888-024-06294-x)
Supplement: Supplementary file 1 — Additional file 1. 1) Search teams for systematic review. 2) Online survey questions. 3) Qualitative interview topic guide. 4) PRISMA diagram – selection of studies for systematic review. 5) Table of characteristics for studies included in systematic review. 6) Reference list for studies included in systematic review. [file 12888_2024_6294_MOESM1_ESM.pdf]

**Additional File 1**

1) Search teams for systematic review
2) Online survey questions
3) Qualitative interview topic guide
4) PRISMA diagram – selection of studies for systematic review
5) Table of characteristics for studies included in systematic review
6) Reference list for studies included in systematic review

**<u>Search terms for systematic review</u>**

**<u>Pubmed and scopus search terms</u>**

**Date filters: 1st Feb 2016 to 1st June 2022**

((("Cognitive behavioural psychotherapy") OR ("Cognitive psychotherapy") OR ("Individual psychotherapy") OR ("Group psychotherapy") OR ("Behavioural psychotherapy")) OR (cognitive therap* OR behavio?r* therap* OR cognitive behavio?r* therap* OR CBT OR psychological therap* OR group therap* OR individual therap* OR dialectical behavio?r* therap* OR DBT OR compassion focus?ed therap* OR compassionate mind training OR CMT OR psychological treatment OR psychological intervention OR mindfulness OR emotion regulation OR acceptance commitment therap* OR ACT OR mindfulness based OR third wave therap* OR third wave cognitive therap*)) AND (((psychiatric inpatient care) OR (acute inpatient mental health care)) OR (mental health AND inpatient care)) AND (acute psychosis OR psychosis OR psychotic OR schizo* OR personality disorder OR PD OR borderline personality disorder OR BPD OR severe mental illness) AND (inpatient OR acute)

# Online Survey

## Terms and Definitions used in Online Survey

| Term | Definition |
|---|---|
| Inpatient | A person who lives in a hospital setting while under treatment. |
| Outcomes | The measures of a treatment/intervention which reflect any changes or results that occur. For example, in a clinical trial of how well psychological therapy works, 'outcomes' might include a quality of life measure or a depression questionnaire. |
| Psychological therapy | This refers to any psychological therapy with a professional/therapist. Psychological therapies are occasionally referred to as talking therapies. They involve exploring psychological difficulties that might be getting in the way of how we would like to feel. Psychological therapy is a collaborative space to explore difficulties in a safe and confidential setting. Psychological therapies can include, but are not limited to, Cognitive Behavioural Therapy, Mindfulness, Interpersonal Therapy, Psychotherapy, Psychodynamic Therapy, and Systemic/Family Therapy. |
| Psychiatric/Acute mental health ward | This refers to a ward in a mental health hospital which people are admitted to voluntarily or whilst they are detained under the Mental Health Act. |

| Group | Question |
|---|---|
| All participants | If a psychological therapy delivered to someone on an acute mental health ward was effective, how would we be able to tell??<br>Can you think of any important outcomes to measure in research assessing the effectiveness of psychological therapy for people on acute mental health wards?<br>If someone is receiving treatment on an acute mental health ward, what kinds of things should change in a positive way for them if the treatment works? |
| Service user | What do you think is the most important aspect of psychological therapy in an acute mental health ward?<br>If you had a psychological therapy during a stay on a mental health ward, what would be the most important things to measure to check whether it had worked or not?<br>Was there any one person/group/intervention which really made a difference for you? If so, what was it? What made it so powerful?<br>Do you have any other feelings or concerns not addressed in previous questions? |
| Carer | What types of improvement would you expect to see in your family member/person you for care for following successful psychological therapy in an acute mental health ward?<br>What are the main ways you would be able to tell your family member/person you care for had got better after receiving a psychological therapy on a mental health ward?<br>What do you consider to be the most important aspects of psychological therapy for your family member/friend in an acute mental health ward? |
| Mental health professional | What do you consider to be the most important aspects of psychological therapy in an acute mental health ward for your service-users?<br>Are there any changes/behaviours you would expect to see in a service-user during or following an effective psychological therapy?<br>What should be the focus of a psychological therapy for someone receiving care on a mental health ward? |
| Researchers | If applicable, what outcomes did you measure in past research (of psychological interventions)?<br>Are there any concepts that you think are important to measure, but chose not to, due to not having a suitable measurement instrument? |
| End users of research | If you were looking to use research to inform changes to the psychological therapies procedure within your professional role, what outcomes would you like to see reported?<br>In your opinion, what measurements would make you think you that a psychological intervention is effective? |

**<u>Development of a Core Outcome Set for psychological therapy trials in acute mental health inpatient services</u>**

**Semi-structured Interview Topic Guide [Service user/carer versions in brackets]**

**1) If a psychological therapy delivered to someone on an acute mental health ward was effective, how would we be able to tell?**

Define 'psychological therapy' and 'psychiatric/acute mental health ward'

- **Psychological therapy** refers to any psychological therapy with a professional/therapist. Psychological therapies are occasionally referred to as talking therapies. They involve exploring psychological difficulties that might be getting in the way of how we would like to feel. Psychological therapy is a collaborative space to explore difficulties in a safe and confidential setting. Psychological therapies can include, but are not limited to, Cognitive Behavioural Therapy, Mindfulness, Interpersonal Therapy, Psychotherapy, Psychodynamic Therapy, and Systemic/Family Therapy.
- **Psychiatric/acute mental health ward** refers to a ward in a mental health hospital which people are admitted to voluntarily or whilst they are detained under the Mental Health Act.

**2) Can you think of any important outcomes to measure in research assessing the effectiveness of psychological therapy for people on acute mental health wards?**

Define 'outcomes'
**Outcomes:** The measures of a treatment/intervention which reflect any changes or results that occur. For example, in a clinical trial of how well psychological therapy works, 'outcomes' might include a quality of life measure or relapse/reoccurrence.

**3) If someone is receiving treatment on an acute mental health ward, what kinds of things should change in a positive way for them if the treatment works?**

**4) What do you think is the most important aspect of psychological therapy in an acute mental health ward? [*Service user]***

**OR**

**What types of improvement would you expect to see in your family member/person you for care for following successful psychological therapy in an acute mental health ward? [*Carer]***

**5) If you had a psychological therapy during a stay on a mental health ward, what would be the most important things to measure to check whether it had worked or not? [*Service user]***

**OR**

**What are the main ways you would be able to tell your family member/person you care for had got better after receiving a psychological therapy on a mental health ward? [*Carer]***

**6) Was there any one person/group/intervention which really made a difference for you? If so, what was it? What made it so powerful? [*Service user]***

**OR**

**What do you consider to be the most important aspects of psychological therapy for your family member/friend in an acute mental health ward? [*Carer]***

**Figure**

*PRISMA Diagram for Systematic Review*

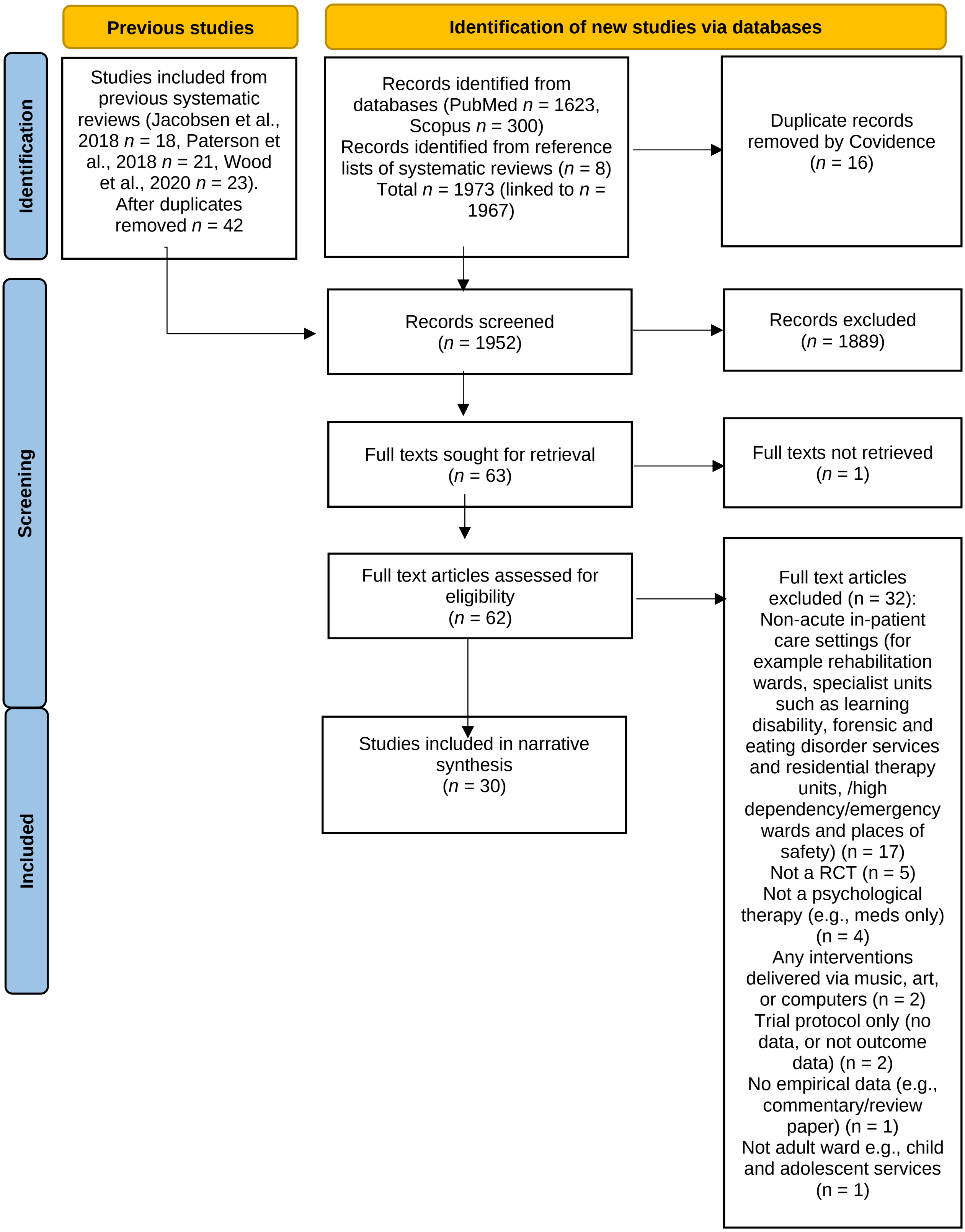

**Table of characteristics for studies included in systematic review (N=30)**

| Study | Location of Trial | Total Sample | Sub-group | Trial Setting | Type of Psychological Intervention | Intervention Method |
|---|---|---|---|---|---|---|
| Aghotor et al. (2010) | Germany | 30 | Diagnosis of a schizophrenic spectrum disorder and patients should have either previously experienced or be currently experiencing delusions | Centre for Psychosocial Medicine, Department of General Psychiatry of the University of Heidelberg (Germany) | Meta-cognitive training | Group |
| Bach et al. (2002) | United States | 80 | Psychotic symptoms | Inpatient | ACT | Individual |
| Bechdolf et al. (2004) | Germany | 88 | Met criteria for an episode of a schizophrenic or related disorder (ICD-10: F 20, F 23, F 25) | Acute inpatient unit | CBT | Group |
| Beutler et al. (1984) | United States | 176 | N/A | Teaching hospital for short-term inpatient care | Other: Three group therapy programs based on an interactive, process-oriented group format, an expressive-experiential-oriented group format; and a behaviourally oriented group format. | Group |

| | | | | | | |
|---|---|---|---|---|---|---|
| Chen et al. (2019) | China | 140 | Bipolar | Acute inpatient | Other: Psychoeducation | Group |
| deRoten et al. (2017) | Switzerland | 153 | DSM IV criteria for unipolar major depressive episode | University Psychiatric Hospital | Psychodynamic | Individual |
| Drury et al. (1996) | United Kingdom | 62 | Screening criteria derived from the WHO first-contact study. Broad spectrum of people presenting with hallucinations, delusions and abnormal behaviour such that a functional psychosis was indicated | Acute inpatient | CBT | Individual; Group |
| Gaudiano and Herbert (2006) | United States | 40 | Psychotic symptoms | Inpatient | ACT | Individual |
| Glick et al. (1985) | United States | 144 | A diagnosis of schizophrenic, schizophreniform disorder, or major affective disorder based on DSM-III | Acute inpatient unit | Other: Family Intervention | Family |
| Graham et al. (2016) | United Kingdom | 59 | Schizophrenia related or bipolar disorder diagnoses, users of community mental health services and also misusing alcohol and/or drugs | Eleven acute wards and three Psychiatric Intensive Care Units | Other: Brief Integrated Motivational Intervention | Individual |
| Habib et al. (2015) | Pakistan | 42 | Diagnosis of schizophrenia according to the DSM-IV-TR | Acute inpatient psychiatry units of three hospitals | CBT | Individual |

| | | | | | | |
|---|---|---|---|---|---|---|
| Haddock et al. (1999) | United Kingdom | 21 | DSM-IV (APA 1994) diagnosis of schizophrenia or schizo-affective disorder confirmed by an independent psychiatrist attached to the project. First treatment for psychosis less than 5 years ago. Currently admitted to an acute general psychiatric ward for onset or relapse of psychotic symptoms | Acute general psychiatric ward | CBT | Individual |
| Hall and Tarrier (2003) | United Kingdom | 25 | A primary diagnosis of a psychotic disorder, experience of auditory hallucinations or delusional ideation and low self esteem, i.e. a score of 132 or less, as assessed on the Robson Self Concept Questionnaire | Inpatient acute NHS psychiatric unit | CBT | Individual |
| Hauschildt et al. (2022) | Germany | 75 | Patients were included if they fulfilled criteria of MDD (i.e., current major depressive single episode, recurrent depression, or dysthymia) verified by the Mini International Neuropsychiatric Interview (Sheehan et al., 1997). | Special unit for depression at a clinic for psychiatry and psychotherapy | Meta-cognitive training | Group |

| | | | | | | |
|---|---|---|---|---|---|---|
| Hayashi et al. (2001) | Japan | 54 | Schizophrenia | Acute inpatient | Other: Intervention based on individual interview sessions, and intended to foster patient attitude and understanding adaptive to the treatment situation by communicating views of illness and treatment in a working relationship formed with patients. | Individual |
| Jacobsen et al. (2020) | United Kingdom | 50 | Diagnosis of schizophrenia-spectrum disorder or psychotic symptoms in the context of an affective disorder (ICD-10 codes F20,39). Reports at least one current positive psychotic symptom (scores > 1 on frequency on self-report symptom scale). | Acute inpatient | Other: Mindfulness-based crisis interventions (MBCI) | Individual |
| Kim et al. (2010) | South Korea | 45 | Diagnosis of schizophrenia confirmed by the Structured Clinical Interview for DSM-IV axis I disorders (SCID-I) | Acute inpatient unit for severe mental illness | Other: Eye Movement Desensitization and Reprocessing (EMDR) | Individual |
| Kumar et al. (2010) | India | 16 | Male patients with the ICD-10 (World Health Organization, 1992) diagnosis of paranoid schizophrenia | Acute inpatient ward | Meta-cognitive training | Group |

| | | | | | | |
|---|---|---|---|---|---|---|
| Lee et al. (2012) | Korea | 25 | Schizophrenia | Acute inpatient | CBT | Individual |
| Lee et al. (2018) | Taiwan | 60 | Patients were enrolled if they met the Diagnostic and Statistical Manual of Mental Disorders (DSM), 4th edition (American Psychiatric Association 1994) criteria for bipolar-I or bipolar-II disorder, were able to be inter- viewed, and had a Hamilton Depression Rating Scale (HDRS) score <17 and a Young Mania Rating Scale (YMRS) score <38 (Keck 2004) to prevent adverse effects caused by patients, mood instability. | Psychiatric acute ward | Other: Brief family-centered care programme | Family |

| | | | | | | |
|---|---|---|---|---|---|---|
| Lu et al. (2012) | China | 126 | Diagnostic criteria for schizophrenia (based on the third edition of the Chinese Classification and Diagnostic Criteria of Mental Disorders), had a duration of illness of at least five years, were clinically stable at the time of enrollment (i.e., total score of Positive and Negative Syndrome Scale [PANSS] <60 or a drop in the total PANSS score of more than 50% after initial treatment of acute symptoms), were 18-65 years of age, and were receiving maintenance treatment with a combination of clozapine and risperidone (the most common combined treatment regimen for chronic patients in our setting). | Acute inpatient | Other: Cognitive remediation therapy | Individual |
| Miller et al., (1989) | United States | 46 | Diagnosis of major depressive disorder according to the Diagnostic Interview Schedule (Robins, Helzer, Croughan & Ratcliff, 1981); b) Beck Depression Inventory (Beck, Ward, Mendelson, Mock & Erbaugh, 1961) > 17; c) Modified Hamilton Rating Scale for Depression 17 item score (Miller, Bishop, Norman & Maddover, 1985) > 17 | Acute inpatient ward | CBT | Individual |

| | | | | | | |
|---|---|---|---|---|---|---|
| Schaub et al. (2016) | Germany | 196 | Diagnosis of schizophrenia-spectrum disorder or other psychotic disorder made by treating psychiatrist according to the Diagnostic and Statistical Manual of Mental Disorders DSM-IV,20 post-acute stage of the illness (ie, remission of acute symptoms) | Department of Psychiatry and Psychotherapy of the Ludwig Maximilian, University at Munich | Other: Group Coping-Oriented Therapy | Group |
| Schramm et al. (2007) | Germany | 130 | Diagnosis of major depressive disorder | Acute psychiatric hospital | Other: Interpersonal Psychotherapy | Individual; Group |
| She et al. (2017) | China | 170 | Diagnosed with Schizophrenia with the Structured Clinical Interview for DSM-IV | Inpatient psychiatric ward | CBT | Individual; Group; Family |
| Sheaves et al. (2018) | United Kingdom | 40 | Self-reported symptoms of insomnia score of 8 on the Insomnia Severity Index (ISI)] | 18-bed male only psychiatric inpatient ward | CBT | Individual |
| Startup et al. (2004) | United Kingdom | 90 | Consecutive admissions to psychiatric hospital, clinical diagnosis of schizophrenia, schizophreniform or schizo-affective disorder, appeared to be suffering an acute psychotic episode, were not already receiving psychological treatment, and showed no evidence of organic mental disorder | Three acute psychiatric hospitals | CBT | Individual |

| | | | | | | |
|---|---|---|---|---|---|---|
| Tyrberg et al. (2017) | Sweden | 22 | Schizophrenia and other psychotic disorders | Psychiatric inpatient unit | ACT | Individual |
| Wood et al. (2018) | United Kingdom | 30 | Met criteria for a schizophrenia-spectrum diagnoses (schizophrenia, schizophreniform disorder, schizoaffective disorder, delusional disorder or psychotic disorder not otherwise specified; ICD-10) or met criteria for an Early Intervention Service (EIS) to allow for diagnostic uncertainty. Self-reported that stigma was causing them negative emotional consequences. | Acute psychiatric wards | CBT | Individual |
| Youssef (1987) | United States | 30 | Affective disorders | Psychiatric unit of a general hospital | Other: Family-patient education programme | Family |

**Reference list for studies included in systematic review (N=30)**


1. Aghotor, J., Pfueller, U., Moritz, S., Weisbrod, M., & Roesch-Ely, D. (2010). Metacognitive training for patients with schizophrenia (MCT): feasibility and preliminary evidence for its efficacy. *Journal of behavior therapy and experimental psychiatry*, *41*(3), 207-211. https://doi.org/10.1016/j.jbtep.2010.01.004
2. Bach, P., & Hayes, S. C. (2002). The Use of Acceptance and Commitment Therapy to Prevent the Rehospitalization of Psychotic Patients: A Randomized Controlled Trial. *Journal of Consulting and Clinical Psychology. 70*(5), 1129–1139. https://doi.org/10.1037//0022-006X.70.5.1129
3. Bechdolf, A., Knost, B., Kuntermann, C., Schiller, S., Klosterkötter, J., Hambrecht, M., & Pukrop, R. (2004). A randomized comparison of group cognitive-behavioural therapy and group psychoeducation in patients with schizophrenia. *Acta Psychiatrica Scandinavica*, *110*(1), 21-28. https://doi.org/10.1111/j.1600-0447.2004.00300.x
4. Beutler, L. E., Frank, M., Schieber, S. C., Calvert, S., & Gaines, J. (1984). Comparative effects of group psychotherapies in a short-term inpatient setting: An experience with deterioration effects. *Psychiatry*, *47*(1), 66-76. https://doi.org/10.1080/00332747.1984.11024227
5. Chen, R., Zhu, X., Capitão, L. P., Zhang, H., Luo, J., Wang, X., Xi, Y., Song, X., Feng, Y., Cao, L., & Malhi, G. S. (2019). Psychoeducation for psychiatric inpatients following remission of a manic episode in bipolar I disorder: a randomized controlled trial. *Bipolar Disorders*, *21*(1), 76-85. https://doi.org/10.1111/bdi.12642
6. deRoten, Y., Ambresin, G., Herrera, F., Fassassi, S., Fournier, N., Preisig, M., & Despland, J. N. (2017). Efficacy of an adjunctive brief psychodynamic psychotherapy to usual inpatient treatment of depression: Results of a randomized controlled trial. *Journal of affective disorders*, *209*, 105-113. https://doi.org/10.1016/j.jad.2016.11.013
7. Drury, V., Birchwood, M., Cochrane, R., & MacMillan, F. (1996). Cognitive therapy and recovery from acute psychosis: A controlled trial: I. Impact on psychotic symptoms. *The British Journal of Psychiatry*, *169*(5), 593-601. https://doi.org/10.1192/bjp.169.5.593
8. Gaudiano, B. A., & Herbert, J. D. (2006). Acute treatment of inpatients with psychotic symptoms using Acceptance and Commitment Therapy: Pilot results. *Behaviour research and therapy*, *44*(3), 415-437. https://doi.org/10.1016/j.brat.2005.02.007
9. Glick, I. D., Clarkin, J. F., Spencer, J. H., Jr, Haas, G. L., Lewis, A. B., Peyser, J., DeMane, N., Good-Ellis, M., Harris, E., & Lestelle, V. (1985). A Controlled Evaluation of Inpatient Family Intervention: I. Preliminary Results of the Six-Month Follow-up. *Archives of General Psychiatry*, *42*(9), 882-886. https://doi.org/10.1001/archpsyc.1985.01790320054007
10. Graham, H. L., Copello, A., Griffith, E., Freemantle, N., McCrone, P., Clarke, L., Walsh, K., Stefanidou, C., Rana, A., & Birchwood, M. (2016). Pilot randomised trial of a brief intervention for comorbid substance misuse in psychiatric in-patient settings. *Acta Psychiatrica Scandinavica*, *133*(4), 298-309. https://doi.org/10.1111/acps.12530
11. Habib, N., Dawood, S., Kingdon, D., & Naeem, F. (2015). Preliminary evaluation of culturally adapted CBT for psychosis (CA-CBTp): findings from developing culturally-sensitive CBT project (DCCP). *Behavioural and Cognitive Psychotherapy*, *43*(2), 200-208. https://doi.org/10.1017/S1352465813000829
12. Haddock, G., Tarrier, N., Morrison, A. P., Hopkins, R., Drake, R., & Lewis, S. (1999). A pilot study evaluating the effectiveness of individual inpatient cognitive-behavioural therapy in early psychosis. *Social Psychiatry and Psychiatric Epidemiology*, *34*(5), 254-258. https://doi.org/10.1007/s001270050141
13. Hall, P. L., & Tarrier, N. (2003). The cognitive-behavioural treatment of low self-esteem in psychotic patients: a pilot study. *Behaviour research and therapy*, *41*(3), 317-332. https://doi.org/10.1016/S0005-7967(02)00013-X

14. Hauschildt, M., Arlt, S., Moritz, S., Yassari, A. H., & Jelinek, L. (2022). Efficacy of metacognitive training for depression as add-on intervention for patients with depression in acute intensive psychiatric inpatient care: A randomized controlled trial. *Clinical Psychology & Psychotherapy*, *29*(5), 1542-1555. https://doi.org/10.1002/cpp.2733
15. Hayashi, N., Yamashina, M., Igarashi, Y., & Kazamatsuri, H. (2001). Improvement of patient attitude toward treatment among inpatients with schizophrenia and its related factors: Controlled study of a psychological approach. *Comprehensive Psychiatry*, *42*(3), 240-246. https://doi.org/10.1053/comp.2001.23136
16. Jacobsen, P., Peters, E., Robinson, E. J., & Chadwick, P. (2020). Mindfulness-based crisis interventions (MBCI) for psychosis within acute inpatient psychiatric settings; a feasibility randomised controlled trial. *BMC Psychiatry*, *20*(1), 193. https://doi.org/10.1186/s12888-020-02608-x
17. Kim, D., Choi, J., Kim, S. H., Oh, D. H., Park, S. C., & Lee, S. H. (2010). A pilot study of brief eye movement desensitization and reprocessing (EMDR) for treatment of acute phase schizophrenia. *Korean Journal of Biological Psychiatry*, *17*(2), 94-102.
18. Kumar, D., Zia Ul Haq, M., Dubey, I., Dotivala, K. N., Veqar Siddiqui, S., Prakash, R., Abhishek, P., & Nizamie, S. H. (2010). Effect of meta-cognitive training in the reduction of positive symptoms in schizophrenia. *European Journal of Psychotherapy & Counselling*, *12*(2), 149-158. https://doi.org/10.1080/13642537.2010.488875
19. Lee, D.-H., Ko, S.-M., Choi, Y.-S., Kim, K.-J., & Park, H. (2012). A Randomized Controlled Pilot Study of Cognitive Behavioral Social Skills Training (Korean version) for Middle- or Older-Aged Patients with Schizophrenia : A Pilot Study. *jkna*, *51*(4), 192-201. https://doi.org/10.4306/jknpa.2012.51.4.192
20. Lee, H.-J., Lin, E. C.-L., Chen, M.-B., Su, T.-P., & Chiang, L.-C. (2018). Randomized, controlled trial of a brief family-centred care programme for hospitalized patients with bipolar disorder and their family caregivers. *International Journal of Mental Health Nursing*, *27*(1), 61-71. https://doi.org/10.1111/inm.12294
21. Lu, H., Li, Y., Li, F., Jiao, X., Shi, W., Guo, K., & Liu, P. (2012). Randomized controlled trial on adjunctive cognitive remediation therapy for chronically hospitalized patients with schizophrenia. *Shanghai Arch Psychiatry*, *24*(3), 149-154. https://doi.org/10.3969/j.issn.1002-0829.2012.03.004
22. Miller, I. W., Norman, W. H., Keitner, G. I., Bishop, S. B., & Dow, M. G. (1989). Cognitive-behavioral treatment of depressed inpatients. *Behavior Therapy*, *20*(1), 25-47. https://doi.org/10.1016/S0005-7894(89)80116-9
23. Schaub, A., Mueser, K. T., von Werder, T., Engel, R., Möller, H.-J., & Falkai, P. (2016). A Randomized Controlled Trial of Group Coping-Oriented Therapy vs Supportive Therapy in Schizophrenia: Results of a 2-Year Follow-up. *Schizophrenia Bulletin*, *42*(suppl_1), S71-S80. https://doi.org/10.1093/schbul/sbw032
24. Schramm, E., van Calker, D., Dykierek, P., Lieb, K., Kech, S., Zobel, I., Leonhart, R., & Berger, M. (2007). An intensive treatment program of interpersonal psychotherapy plus pharmacotherapy for depressed inpatients: acute and long-term results. *American Journal of Psychiatry*, *164*(5), 768-777. https://doi.org/10.1176/ajp.2007.164.5.768
25. She, S., Deng, Y., Chen, Y., Wu, C., Yi, W., Lu, X., Chen, X., Li, J., Li, R., Zhang, J., Xiao, D., Wu, H., Ning, Y., & Zheng, Y. (2017). Two-stage integrated care versus antipsychotic medication alone on outcomes of schizophrenia: One-year randomized controlled trial and follow-up. *Psychiatry Research*, *254*, 164-172. https://doi.org/https://doi.org/10.1016/j.psychres.2017.04.054
26. Sheaves, B., Freeman, D., Isham, L., McInerney, J., Nickless, A., Yu, L.-M., Rek, S., Bradley, J., Reeve, S., Attard, C., Espie, C. A., Foster, R., Wirz-Justice, A., Chadwick, E., & Barrera, A. (2018). Stabilising sleep for patients admitted at acute crisis to a psychiatric hospital (OWLS):

an assessor-blind pilot randomised controlled trial. *Psychological medicine*, *48*(10), 1694-1704. https://doi.org/10.1017/S0033291717003191
27. Startup, M., Jackson, M. C., & Bendix, S. (2004). North Wales randomized controlled trial of cognitive behaviour therapy for acute schizophrenia spectrum disorders: outcomes at 6 and 12 months. *Psychological Medicine*, *34*(3), 413-422. https://doi.org/Doi 10.1017/S0033291703001211
28. Tyrberg, M. J., Carlbring, P., & Lundgren, T. (2016). Brief acceptance and commitment therapy for psychotic inpatients: A randomized controlled feasibility trial in Sweden. *Nordic Psychology*, 1-16. https://doi.org/10.1080/19012276.2016.1198271
29. Wood, L., Byrne, R., Enache, G., & Morrison, A. P. (2018). A brief cognitive therapy intervention for internalised stigma in acute inpatients who experience psychosis: A feasibility randomised controlled trial. *Psychiatry Research*, *262*, 303-310. https://doi.org/10.1016/j.psychres.2017.12.030
30. Youssef, F. (1987). Discharge planning for psychiatric patients: the effects of a family-patient teaching programme. *Journal of Advanced Nursing*, *12*(5), 611-616. https://doi.org/10.1111/j.1365-2648.1987.tb03052.x
